# A Retrospective Multicenter Study of Risk Factors, Stratification, and Prognosis of Lymph Node Metastasis in T1 and T2 Colorectal Cancer

**DOI:** 10.3390/jcm12247744

**Published:** 2023-12-18

**Authors:** Eui Myung Kim, Il Tae Son, Byung Chun Kim, Jun Ho Park, Byung Mo Kang, Jong Wan Kim

**Affiliations:** 1 Department of Surgery, Dongtan Sacred Heart Hospital, Hallym University College of Medicine, 40, Sukwoo-Dong, Hwaseong-si 445-170, Republic of Korea; wishnhope@hallym.or.kr; 2 Department of Surgery, Hallym Sacred Heart Hospital, Hallym University College of Medicine, Anyang-si 445-907, Republic of Korea; 1tae99@hallym.or.kr; 3 Department of Surgery, Kangnam Sacred Heart Hospital, Hallym University College of Medicine, 948-1, 1, Shingil-ro, Yeongdeungpo-gu, Seoul 150-950, Republic of Korea; bckimgs@hallym.or.kr; 4 Department of Surgery, Kangdong Sacred Heart Hospital, Hallym University College of Medicine, 445 Gil-1-dong, Gangdong-gu, Seoul 134-701, Republic of Korea; zooono@hanmail.net; 5 Department of Surgery, Chun Cheon Sacred Heart Hospital, Hallym University College of Medicine, 77 Sakju-ro, Chuncheon-si 200-130, Republic of Korea; kbm0728@hallym.or.kr

**Keywords:** colorectal cancer, lymph node metastasis, risk factor

## Abstract

Background. The objective of this study was to compare the long-term prognosis of patients with T1 and T2 colorectal cancer (CRC) according to lymph node metastasis (LNM) and to identify risk factors for LNM. Methods. We retrospectively reviewed patients who underwent curative resection for T1 or T2 CRC at five University-affiliated hospitals between January 2012 and December 2021. The patients were divided into several groups depending on the presence of LNM or the number of risk factors. Results. Of the total 765 patients, 87 (11.3%) patients had LNM. These patients had poorer recurrence-free survival (RFS) than patients without LNM (72.6% vs. 88.6%). The multivariable analysis showed that high-grade tumors (*p* = 0.003), lymphovascular invasion (*p* < 0.001), and rectal location (*p* = 0.049) were independent predictors of LNM. When divided into groups according to the number of the three risk factors, the risk of LNM increased from 5.4% (ultralow-risk group; no risk factor) to 60.0% (high-risk group; all three risk factors) and the 5-year RFS rate decreased from 96.3% in the ultralow-risk group to 60% in the high-risk group (*p* < 0.001). Conclusion. Radical surgery should be considered for T1 and T2 CRC patients with these risk factors.

## 1. Introduction

Tumors confined to the muscularis propria (T1 and T2) are usually considered early cancers and are likely to be cured by complete resection of the tumor [1,2]. Implementation of population-based screening programs and advances in endoscopic techniques have led to increased numbers of patients being diagnosed with early colorectal cancer (CRC) [3]. Among tumors within the muscularis propria, lymph node metastasis (LNM) was found in 4.7–12.3% of patients with T1 early CRC [4,5,6,7,8,9,10,11,12,13,14], increasing to 18.0–24.3% in patients with T2 cancers [1,2,7,9,15].

The most appropriate management of early CRC remains controversial. Endoscopic techniques, including endoscopic submucosal dissection and endoscopic mucosal resection, are commonly performed in early CRC and are associated with fewer complications, a shorter hospital stay, and a quicker recovery than surgery [16]. However, there is a risk of recurrence after endoscopic resection owing to residual tumor and LNM. Although patients with early CRC often undergo radical surgery because of the risk of LNM, possible complications of this procedure include infection, anastomosis leakage, and mortality [17]. Moreover, considering the rate of LNM in T1 or T2 CRC, about 80–90% of patients without LNM could undergo unnecessary radical surgery.

Thus, identification of the clinical and histopathological features associated with the risk of LNM is important for choosing the most appropriate treatment modality for early CRC. Several previous systematic reviews and meta-analyses reported that a depth of submucosal invasion >1000 µm, poorly differentiated tumors, lymphovascular invasion (LVI), and tumor budding were risk factors for LNM [18,19,20,21,22]. According to the Korean clinical practice guidelines, risk factors for LNM in early CRC include a poor histologic type, deep submucosal invasion, LVI, and tumor budding, and additional surgery is recommended if any of these risk factors are listed in the pathologic report [23]. In a recent study, the authors predicted LNM in T1 CRC based on Tn (a type of tumor-associated carbohydrate antigen) as a molecular marker [24].

The aim of the present study was to compare the long-term prognosis of patients with T1 and T2 CRC according to LNM and to identify risk factors for LNM.

## 2. Methods

### 2.1. Study Population

We performed a retrospective review of the medical records of patients who underwent curative resection for T1 or T2 CRC at five Hallym University-affiliated hospitals (Dong Tan Sacred Heart Hospital, Hallym Sacred Heart Hospital, Gang Nam Sacred Heart Hospital, Chun Cheon Sacred Heart Hospital, and Gang Dong Sacred Heart Hospital) between January 2012 and December 2021. Patients underwent surgery as initial treatment after biopsy or additional radical resection with lymphadenectomy after endoscopic resection according to the state of the resection margin and the depth of invasion described in the histopathology reports. Patients with familial adenomatous polyposis (FAP) or hereditary nonpolyposis colorectal cancer (HNPCC) and those with recurrent disease or stage IV cancer were excluded. Patients who underwent neoadjuvant chemoradiation therapy, patients diagnosed with non-adenocarcinoma (neuroendocrine tumor, gastrointestinal tumor, and sarcoma), and patients with incomplete medical records were also excluded.

### 2.2. Data Collection

Patient characteristics, histologic variables, and oncologic outcomes were retrieved from the medical records. The patient characteristics consisted of age, gender, body mass index (BMI), American Society of Anesthesiologists (ASA) score, tumor location, and whether endoscopic resection was attempted. Information retrieved from the histopathology reports included the presence of LNM, histologic cancer grade, number of harvested lymph nodes, tumor size, LVI, perineural invasion (PNI), and tumor stage according to the eighth edition of the American Joint Commission on Cancer (AJCC) TNM staging system [25].

The tumor location was classified into three groups: right colon, left colon, or rectum. Right and left colon cancers were combined in the analysis. Right colon cancer was defined as a cancer located between the cecum and the transverse colon. Left colon cancer was defined as a cancer located between the splenic flexure and the sigmoid colon. Histological differentiation was based on World Health Organization guidelines [26]. In this study, well and moderately differentiated adenocarcinomas were considered low-grade tumors, and poorly differentiated and undifferentiated adenocarcinomas were considered high-grade tumors. We stratified the T1 or T2 CRC patients according to the number of risk factors identified in the multivariable analyses for LNM.

### 2.3. Follow-Up

Patients underwent physical examinations and laboratory tests such as cancer antigen 19-9 and carcinoembryonic antigen (CEA) every 3–6 months for the first 2 years and every 6 months thereafter until 5 years after treatment. Chest and abdominopelvic computed tomography (CT) scans were conducted every 6 months until 5 years after treatment. Colonoscopy was performed after 1 year and then biennially during the follow-up period.

### 2.4. Oncologic Outcome and Objectives

We evaluated the long-term oncologic outcomes in terms of recurrence-free survival (RFS), which is calculated as the time from the date of tumor resection to the date of recurrence, death from any cause, or the last follow-up.

The primary objective of this study was to compare the 5-year RFS according to the presence of LNM in patients with T1 and T2 CRC. Our secondary objectives were to identify risk factors associated with LNM and to compare the 5-year RFS according to the number of risk factors identified in the multivariable analyses for LNM.

### 2.5. Statistical Analysis

All statistical analyses were performed using SPSS version 26.0 (IBM, Armonk, NY, USA). Categorical variables are presented as the number and percent of patients and were analyzed using Fisher’s exact test or the χ^2^ test. Continuous variables are presented as the mean and standard deviation and were compared with the Mann–Whitney U test or Student’s *t* test. RFS was analyzed using the Kaplan–Meier method, and differences were compared using the log-rank test. Cox’s proportional hazards regression model was used to identify risk factors for RFS. The factors included in the multivariable analysis were age (≥65 years), gender (men), ASA (≥3), histological grade (poor/undifferentiated), T2 stage, PNI, LVI, tumor size (≥2.4 cm), endoscopic resection, LNM, and tumor location (rectal cancer vs. all colon cancer), which were previously reported to be associated with recurrence. Multivariable logistic regression was performed to identify independent predictors of LNM. The variables included in the multivariable analysis were the same as those used in Cox’s proportional hazards regression model, except for LNM. Values of *p* < 0.05 were considered statistically significant.

## 3. Results

### 3.1. Patient Disposition

A total of 956 patients with T1 or T2 CRC underwent surgery at the five University-affiliated hospitals during the 10-year study period. We excluded patients with synchronous CRC (*n* = 42), patients with neuroendocrine tumors or gastrointestinal tumors (*n* = 28), patients with incomplete medical records (*n* = 18), patients with FAP or HNPCC (*n* = 5), and patients who underwent neoadjuvant chemoradiotherapy before surgery (*n* = 98). After excluding these 191 patients, 765 patients were included in the study, of which 87 were included in the LNM (+) group (11.4%) and 678 in the LNM (−) group (88.6%).

### 3.2. Patients’ Characteristics

The mean ages of the LNM (+) and LNM (−) groups were 65.3 and 65.6 years, respectively (*p* = 0.857) (Table 1). The proportions of men/women, BMI, ASA score, and presence of comorbidities were similar in both groups. However, the proportion of patients with rectal cancer was greater in the LNM (+) group than in the LNM (−) group (49.4% vs. 34.84%, *p* = 0.008).

### 3.3. Histopathological Outcomes

There were no differences between the two groups in terms of tumor size, number of harvested lymph nodes, and proportion of patients with ≥12 harvested lymph nodes (Table 2). The proportions of patients with LVI (56.3% vs. 15.6%, *p* < 0.001), PNI (10.3% vs. 2.8%, *p* < 0.001), and T2 cancer (58.6% vs. 38.9%, *p* < 0.001) were greater in the LNM (+) group than in the LNM (−) group. The proportion of patients with high-grade tumors was also higher in the LNM (+) group than in the LNM (−) group (10.3% vs. 2.8%, *p* = 0.001).

### 3.4. Prognosis According to the Presence of LNM

**Figure 1:**
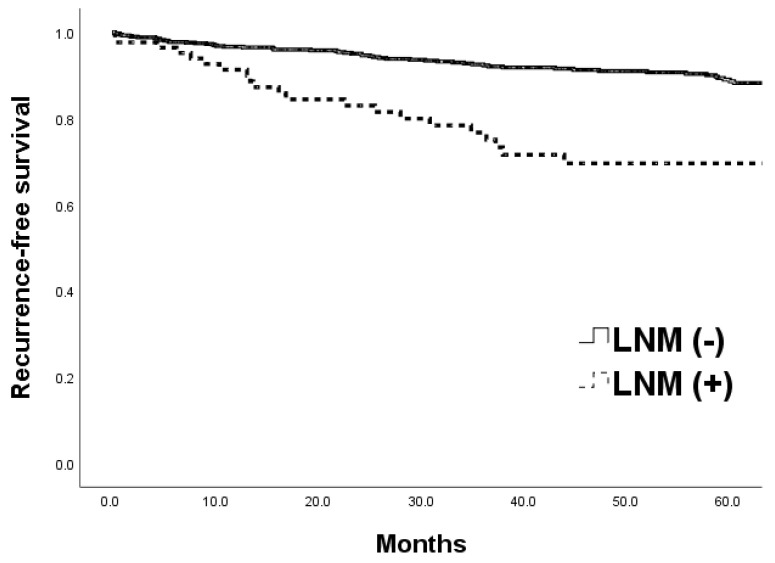
Comparison of 5-year recurrence-free survival curves of patients with T1 or T2 colorectal cancer between lymph node metastasis (LNM) (+) and LNM (−) groups (72.6% vs. 88.6%, *p* < 0.001).

The mean duration of follow-up was 52.4 months overall (range 2–138 months), 48.4 months in the LNM (+) group, and 53.1 months in the LNM (−) group. The 5-year RFS rate was lower in the LNM (+) group than in the LNM (−) group (72.6% vs. 88.6%, *p* < 0.001; Figure 1).

### 3.5. Factors Affecting Prognosis

In a univariate analysis, factors associated with poorer RFS were age ≥ 65 years (*p* < 0.001), ASA ≥ 3 (*p* < 0.001), T2 stage (*p* = 0.020), LVI (*p* < 0.001), CEA ≥ 5 ng/mL (*p* < 0.001), and LNM (*p* < 0.001) (Table 3). In the multivariable analysis, factors associated with poorer RFS were age ≥ 65 years (*p* = 0.015), ASA ≥ 3 (*p* = 0.029), LVI (*p* = 0.043), CEA ≥ 5 ng/mL (*p* = 0.006), and LNM (*p* = 0.012).

### 3.6. Risk Factors for LNM

Considering the significant association between LNM and RFS, we performed univariate and multivariable analyses to identify possible risk factors for LNM (Table 4). The univariate analyses showed that high-grade tumors (*p* = 0.001), T2 stage (*p* = 0.001), LVI (*p* < 0.001), PNI (*p* = 0.001), and rectal location (*p* = 0.049) were associated with LNM in patients with CRC. The multivariable analysis showed that high-grade tumors (*p* = 0.003), LVI (*p* < 0.001), and rectal location (*p* = 0.049) were independent predictors of LNM (Table 4).

### 3.7. Prognosis According to the Presence of Risk Factors for LNM

**Figure 2:**
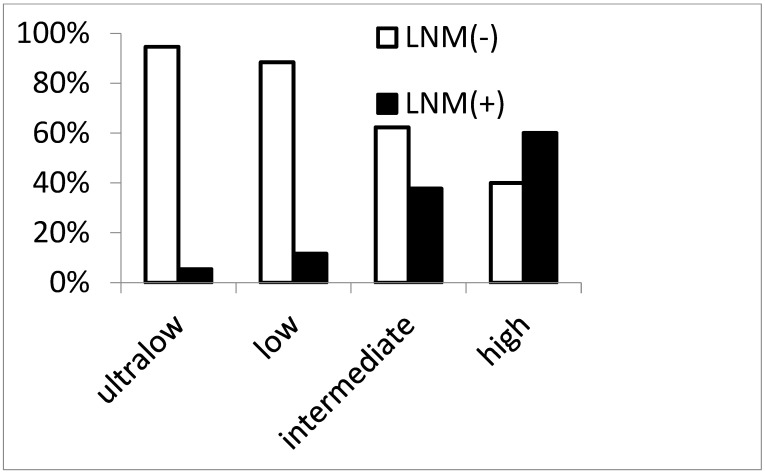
Rate of lymph node metastasis in groups according to the number of risk factors (ultralow-risk group (no risk factor), 5.4%; low-risk group (one risk factor), 11.6%; intermediate-risk group (two risk factors), 37.5%; high-risk group (three risk factors), 60%; *p* < 0.001).

Figure 2 shows the rate of LNM in groups of patients stratified by the number of risk factors identified in the multivariable regression analysis (high-grade tumor, LVI, and rectal location).

**Figure 3:**
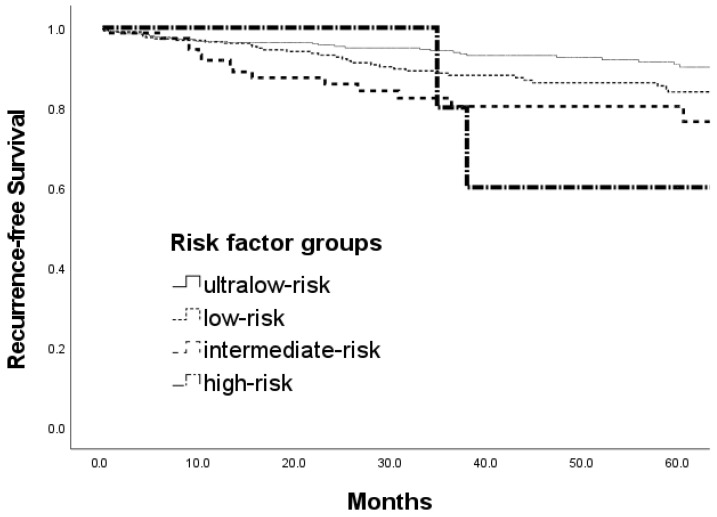
Comparison of 5-year recurrence-free survival rates of patients with T1 or T2 colorectal cancer according to the number of risk factors (ultralow-risk group (no risk factor), 96.3%; low-risk group (one risk factor), 94.5%; intermediate-risk group (two risk factors), 76.5%; high-risk (three risk factors), 60.0%; *p* < 0.001).

Of the 87 patients with LNM, the rate of metastasis increased from 5.4% (21/391) in the ultralow-risk group (no risk factors) to 11.6% (34/292) in the low-risk group (one risk factor), 37.5% (29/77) in the intermediate-risk group (two risk factors), and 60% (3/5) in the high-risk group (three risk factors) (*p* < 0.001). Among these four groups, the 5-year RFS rate was greatest in the ultralow-risk group (ultralow-risk: 96.3%; low-risk: 94.5%; intermediate-risk: 76.5%; high-risk: 60.0%; *p* < 0.001; Figure 3).

## 4. Discussion

In the present study, 87 (11.3%) patients had LNM. These patients showed more aggressive histological features (higher proportion of patients with poorly or undifferentiated adenocarcinoma, LVI, and rectal cancer) and poorer RFS than patients without LNM. When divided into groups according to the number of these three risk factors identified in our study, the risk of LNM increased from 5.4% (ultralow-risk group; no risk factors) to 60.0% (high-risk group; all three risk factors), and the RFS decreased from 96.3% (ultralow-risk group) to 60% (high-risk group).

The present study showed that the incidence of LNM among these patients with T1 or T2 cancer was 11.4% (8% in T1 and 16.2% in T2), which is consistent with the rates of 12.7% to 21% reported in previous studies (4.7–17.6% for T1 and 18.0–24.5% for T2) [1,2,7,9,13,14,15]. LNM is well known as an important risk factor for CRC and is hence included in the assessment of tumor stage [25]. Previous studies have reported that the presence of LNM in patients with T1 or T2 CRC affects the prognosis, including 5-year overall survival, 5-year disease-free survival, and 5-year cancer-specific survival [2,7,9]. Therefore, it is necessary to identify potential risk factors for LNM to help select the most suitable treatment modality. Patients in the low-risk group can undergo endoscopic resection without unnecessary surgery, whereas patients in the high-risk group should undergo radical resection and lymphadenectomy according to oncological criteria.

Many previous studies have investigated tumor location as a predictive factor for LNM. Some studies reported a higher rate of LNM for cancers located in the rectum than those located in the colon [9,11], whereas others did not find an association between tumor location and metastasis [4,5,6,8,10,14]. One possible reason for these discrepancies is that some studies divided the tumor location into three, five, or eight categories [5,6,10,14] instead of two categories (colon and rectum). Another possible explanation is that most of the studies excluded concurrent chemoradiotherapy, but some studies did not mention whether it was excluded [5]. In a systematic review of 10 studies with 2722 patients, rectal cancer was a risk factor for LNM compared with colon cancer (LNM rate: 13.8% vs. 9.9%, 95% confidence interval (CI) 1.1–1.7, *p* < 0.001) [19]. In the present study, rectal cancer was associated with increased risk of LNM in patients with T1 and T2 CRC (*p* = 0.049).

In many previous studies, LVI has been reported as an important and decisive risk factor for LNM [1,2,4,6,13,14,15]. Moreover, five meta-analyses concluded that LVI is an important risk factor for LNM [18,19,20,21,22]. In one of these meta-analyses, Glasgow et al. performed a systematic review of 76 studies with 42 histopathological features of early CRC and reported that LVI was the most important factor (odds ratio (OR) 8.62, 95% CI 7.55–9.84, *p* = 0.003), followed by tumor budding (OR 5.75), tumor depth (OR 2.62), and tumor differentiation (OR 2.38) [20]. The present study showed that LVI was a significant predictive factor for LNM in the multivariable analysis (OR 6.161, 95% CI 3.560–10.662, *p* < 0.001). By comparison, some studies have questioned whether LVI is an important factor in LNM [27,28]. However, those studies consisted of 47 and 182 patients, and these numbers are too low to draw definitive conclusions [27,28].

The histologic grade of the tumor was reported to be a risk factor for LNM in early CRC [4,8,9,10,11,12,13,14]. In a recent meta-analysis of 23 cohort studies comprising 4510 patients, poor differentiation was a risk factor for LNM (OR 5.60, 95% CI 2.90–10.82), and its OR was greater than that for LVI (OR 4.81) and depth of submucosal invasion > 1 mm (OR 3.87) [18]. However, previous studies reported that high-grade differentiation was not a risk factor for LNM [2,7]. In the present study, high-grade tumors were significantly associated with LNM in the multivariable analysis (OR 3.8, 95% CI 1.47–9.8, *p* = 0.006). These inconsistencies may be explained by the low proportion of patients with high-grade histology, ranging from 2.1% to 5.7% in most previous studies except one (17.6%) [2,4,7,8,9,10,11,12,14]. Yosida et al. reported that, among 97 patients with lymphatic or venous invasion, LNM was not present in 29 patients with pure well-differentiated adenocarcinoma (PWDA), but LNM was found in 14.7% (10 of 68) of patients with non-PWDA (*p* = 0.029), suggesting that PWDA is a novel histological factor for reduced risk of LNM in patients with CRC [9].

Several studies have combined several risk factors for LNM and compared the prognosis between groups of patients [1,5,6,7,10,12,13]. Huh et al. identified lymphatic invasion, vascular invasion, and tumor budding as risk factors for LNM and categorized the patients into no-risk, low-risk, and high-risk groups based on those risk factors [1]. The rate of LNM (*p* < 0.001) and the 5-year DFS rate (*p* < 0.001) differed significantly among the three groups. A nomogram using clinicopathological factors obtained from a multivariable logistic regression model was also reported to be effective for predicting LNM [8,29]. Oh et al. established a new model for predicting the probability of LNM that was based on five pathological variables: deep submucosal invasion, vascular invasion, tumor budding, high-grade histology, and background adenoma (BGA). They reported that the probability of LNM ranged from 1.2% to 83.5% for 32 combinations of these variables [8]. The present study showed the rate of LNM increased from 5.4% in the ultralow-risk group to 60% (3/5) in the high-risk group (*p* < 0.001), and the 5-year RFS decreased from 96.5% to 60.0% (*p* = 0.002). Furthermore, LNM occurred in some patients in the ultralow-risk group, despite the absence of the three risk factors. These results suggest that there are other risk factors for LMN, including some that were not evaluated in our study, such as tumor budding and BGA. Future studies including other factors are needed to investigate these further.

There are several limitations to the present study. Because this study was performed retrospectively, several histological features relevant to LNM were not recorded in the medical records or were excluded from the present study because they were recorded later. Second, although the present study included a larger number of patients than previous studies [1,2,9,13,14], the number of patients may be insufficient to comprehensively evaluate risk factors for LNM. Third, the present study only included patients who underwent surgery to allow evaluation of the presence of LNM in resected specimens. Therefore, care should be taken when applying the results of the risk factors identified in this study to patients who underwent endoscopic resection without surgery. For these reasons, a prospective study of a large number of patients and other histological features is required to assess risk factors for LNM in T1 and T2 CRC and obtain important data to help guide the most appropriate treatment plan, including radical surgery, adjuvant chemotherapy, and radiotherapy. Moreover, in the eighth edition of the AJCC TNM staging system [25], the N stage is assigned according to the number of metastatic lymph nodes. A recent study also demonstrated that the prognosis may vary depending on the extent of LNM (D1 vs. D2 + D3) [30]. Therefore, further research is needed to investigate the distribution of LNM and its associated risk factors. Despite these limitations, we believe that this study provides valuable information about risk factors for LNM in patients with T1 and T2 CRC, as well as the prognosis of patients stratified by these risk factors.

## 5. Conclusions

The present study indicates that LVI, high-grade differentiation, and rectal cancer are significant risk factors for LNM in patients with T1 and T2 CRC. In addition, patients with more risk factors had poorer RFS. Therefore, it is reasonable to consider radical surgery for patients with these risk factors after primary endoscopic resection. Moreover, in cases undergoing primary surgery without endoscopic resection, it is reasonable to use an active adjuvant treatment based on the pathology results.

## Figures and Tables

**Table 1:** Patient characteristics according to the presence of lymph node metastasis.

	LNM (−) (*n* = 678)	LNM (+) (*n* = 87)	*p*
Age (years)	65.6 (10.9)	65.3 (10.4)	0.857
Gender			0.566
Men	259 (38.2)	36 (41.4)	
Women	419 (61.8)	51 (58.6)	
BMI (kg/m^2^)	24.0 (3.4)	24.2 (3.4)	0.562
CEA	3.2 (3.1)	3.0 (2.4)	0.724
ASA			0.216
I	82 (12.1)	3 (3.4)	
II	386 (56.9)	57 (65.5)	
III/IV/V	210 (31.0)	27 (31.0)	
Comorbidities	487 (71.8)	62 (71.3)	0.912
Comorbidities ≥ 2	247 (36.4)	34 (39.1)	0.629
Location of tumor			0.008
Right colon	192 (28.3)	16 (18.4)	
Left colon	250 (36.9)	28 (32.2)	
Rectum	236 (34.8)	43 (49.4)	
Endoscopic resection	139 (20.5)	13 (14.9)	0.221

Data are presented as the number of patients (%) or mean (standard deviation) unless otherwise stated. LNM; lymph node metastasis, *n*; number, BMI; body mass index, CEA; carcinoembryonic antigen, ASA; American Society of Anesthesiologists.

**Table 2:** Pathological outcome according to the presence of lymph node metastasis.

	LNM (−) (*n* = 678)	LNM (+) (*n* = 87)	*p*
Histologic type, *n* (%)			<0.001
Well	299 (44.1)	18 (20.7)	
Moderate	360 (53.1)	60 (69.0)	
Poorly/Undifferentiated	19 (2.8)	9 (10.3)	
Tumor size (cm)	2.4 (1.6)	2.3 (1.2)	0.515
Tumor size ≥ 3 cm	214 (31.7)	21 (24.1)	0.150
LVI	105 (15.6)	49 (56.3)	<0.001
PNI	19 (2.8)	9 (10.3)	<0.001
*n* of harvested LN	18.5 (13.2)	18.5(9.3)	0.979
LN ≥ 12	575 (84.8)	75 (86.2)	0.731
T			<0.001
T1	414 (61.6)	36 (41.4)	
T2	264 (38.9)	51 (58.6)	

Data are presented as the number of patients (%) or mean (standard deviation) unless otherwise stated. LNM; lymph node metastasis, LVI; Lymphovascular invasion, PNI; Perineural invasion, *n*; number, LN; lymph nodes.

**Table 3:** Univariate and multivariate analysis of recurrence-free survival.

Variable	Univariate Analysis		Multivariate Analysis	
	OR (95% CI)	*p*	OR (95% CI)	*p*
Age ≥ 65 (years)	2.434 (1.560–3.797)	<0.001	1.845 (1.123–3.028)	0.015
Men	0.834 (0.545–1.278)	0.405	0.890 (0.572–1.384)	0.604
ASA ≥ 3	2.257 (1.496–3.405)	<0.001	1.668 (1.055–2.638)	0.029
Poor/undifferentiated	1.300 (0.477–3.543)	0.608	1.106 (0.392–3.116)	0.849
T2	1.628 (1.081–2.452)	0.020	1.199 (0.704–2.041)	0.505
LVI	2453 (1.606–3.748)	<0.001	1.724 (1.017–2.923)	0.043
PNI	0.803 (0.254–2.538)	0.709	0.516 (0.155–1.718)	0.281
Tumor size ≥ 2.4 cm	1.210 (0.801–1.827)	0.365	0.840 (0.518–1.361)	0.478
CEA ≥ 5 ng/mL	2.464 (1.530–3.968)	<0.001	2.009 (1.217–3.318)	0.006
Endoscopic resection	0.634 (0.338–1.192)	0.157	0.731 (0.351–1.525)	0.404
LN metastasis	2.615 (1.606–4.257)	<0.001	2.094 (1.183–3.707)	0.012
Rectum	1.261 (0.832–1.909)	0.274	1.188 (0.763–1.851)	0.446

OR; Odds Ratio, CI; Confidence interval, ASA; American Society of Anesthesiologists, LVI; Lymphovascular invasion, PNI; Perineural invasion, CEA; carcinoembryonic antigen, LN; lymph node.

**Table 4:** Univariate and multivariate analysis of lymph node metastasis.

Variable	Univariate Analysis		Multivariate Analysis	
	OR (95% CI)	*p*	OR (95% CI)	*p*
Age ≥ 65 (years)	1.120 (0.715–1.754)	0.621	0.997 (0.584–1.702)	0.991
Men	1.142 (0.725–1.798)	0.567	1.105 (0.662–1.844)	0.704
ASA ≥ 3	0.956 (5.88–1.557)	0.858	1.029 (0.582–1.819)	0.922
Poor/undifferentiated	4.002 (1.750–9.151)	0.001	3.793 (1.469–9.789)	0.003
T2	2.222 (1.411–3.497)	0.001	1.611 (0.873–2.973)	0.127
LVI	7.000 (4.366–11.223)	<0.001	6.161 (3.560–10.662)	<0.001
PNI	3.960 (1.731–9.065)	0.001	1.154 (0.441–3.021)	0.771
Tumor size ≥ 2.4 cm	1.007 (0.641–1.581)	0.978	0.592 (0.336–1.044)	0.070
CEA ≥ 5 ng/mL	0.702 (0.340–1.449)	0.338	0.540 (0.246–1.184)	0.124
Endoscopic resection	0.681 (0.367–1.264)	0.224	1.286 (0.604–2.736)	0.514
Rectum	1.830 (1.168–2.868)	0.008	1.976 (1.003–3.443)	0.049

OR; Odd Ratio, CI; Confidence interval, ASA; American Society of Anesthesiologists, LVI; Lymphovascular invasion, PNI; Perineural invasion, CEA; carcinoembryonic antigen.

## Data Availability

The data underlying this article will be shared upon reasonable request to the corresponding author.
